# Differentially Expressed MiRNAs and tRNA Genes Affect Host Homeostasis During Highly Pathogenic Porcine Reproductive and Respiratory Syndrome Virus Infections in Young Pigs

**DOI:** 10.3389/fgene.2019.00691

**Published:** 2019-08-02

**Authors:** Damarius S. Fleming, Laura C. Miller

**Affiliations:** ^1^ORAU/ORISE, Oak Ridge, TN, United States; ^2^Virus and Prion Diseases of Livestock Research Unit, National Animal Disease Center, USDA, Agricultural Research Service, Ames, IA, United States.

**Keywords:** miRNA—microRNA, tRNA, differential gene expression, porcine reproductive and respiratory syndrome virus, whole blood, pigs (*Sus scrofa*)

## Abstract

**Background:** Porcine respiratory and reproductive syndrome virus (PRRSV) is a single-stranded RNA virus member that infects pigs and causes losses to the commercial industry reaching upward of a billion dollars annually in combined direct and indirect costs. The virus can be separated into etiologies that contain multiple heterologous low and highly pathogenic strains. Recently, the United States has begun to see an increase in heterologous type 2 PRRSV strains of higher virulence (HP-PRRSV). The high pathogenicity of these strains can drastically alter host immune responses and the ability of the animal to maintain homeostasis. Because the loss of host homeostasis can denote underlying changes in gene and regulatory element expression profiles, the study aimed to examine the effect PRRSV infections has on miRNA and tRNA expression and the roles they play in host tolerance or susceptibility.

**Results:** Using transcriptomic analysis of whole blood taken from control and infected pigs at several time points (1, 3, 8 dpi), the analysis returned a total of 149 statistically significant (FDR ⫹ 0.15) miRNAs (n = 89) and tRNAs (n = 60) that were evaluated for possible pro- and anti-viral effects. The tRNA differential expression increased in both magnitude and count as dpi increased, with no statistically significant expression at 1 dpi, but increases at 3 and 8 dpi. The most abundant tRNA amino acid at 3 dpi was alanine, while glycine was the most abundant at 8 dpi. For the miRNAs, focus was put on upregulation that can inhibit gene expression. These results yielded candidates with potential anti- and pro-viral actions such as Ssc-miR-125b, which is predicted to limit PRRSV viral levels, and Ssc-miR-145-5p shown to cause alternative macrophage priming. The results also showed that both the tRNAs and miRNAs displayed expression patterns.

**Conclusions:** The results indicated that the HP-PRRSV infection affects host homeostasis through changes in miRNA and tRNA expression and their subsequent gene interactions that target and influence the function of host immune, metabolic, and structural pathways.

## Introduction

Porcine respiratory and reproductive syndrome virus (PRRSV) is a single-stranded RNA virus member of the Nidovirales order that infects pigs and causes losses to the commercial swine industry that reach upward of a billion dollars annually in combined (Holtkamp et al., 2013) direct and indirect costs. The virus can be separated into etiologies that contain multiple heterologous strains due to the high mutation and recombination rates observed within the virus, leading to the evolution of both low and highly pathogenic PRRSV strains. Within industry herds, the low pathogenic strains can cause persistent infections that can last the entirety of the pigs “commercial life,” while the highly pathogenic strains often cause acute illness and increased mortality. Although PRRSV infections can be found globally, a highly pathogenic Chinese type 2 PRRSV strain, not present in the United States, has ravaged much of Asia by presenting as an acute infection leading to high mortality in animals early in the commercial process. Additionally, recent studies have observed that the United States has begun to see an increase in heterologous type 2 PRRSV strains of increased virulence (van Geelen et al., 2018). The high pathogenicity of these strains can drastically alter host immune responses and the ability of the animal to maintain homeostasis. Because changes in the homeostasis of an individual can denote underlying changes in health and gene expression profiles, researchers have conducted studies to evaluate the host–virus interaction in the attempt to better understand the genetics involved in the host immune response to PRRSV (Shanmukhappa et al., 2007; Miller et al., 2012; Xiao et al., 2015; Miller et al., 2017).

The health of an animal, prior to and after illnesses, is, in many ways, a measure of the ability of that individual to maintain or return to a homeostatic state in which the interplay between immunologic and metabolic responses are in balance (Hotamisligil, 2006). In livestock, this balance is crucial to the health and growth of the animal. This change in internal balance of the host is most evident at the whole animal level, in which clinical signs of an illness can be represented by phenotypes such as fever, lameness, and changes in growth. However, these changes start at the genomic level where dysregulation can be represented as perturbations in the host ability to maintain proper communication between cellular receptors and signaling. This change in signaling cascades facilitates invasion of microbes, like PRRSV viral particles, into the host system as the miscommunication ameliorates pattern recognition receptor (PRR) evasion by the virus (Bowie and Unterholzner, 2008). The cell tropism of PRRSV, in general, is the monocyte-derived cells of the innate arm of immunity that become pulmonary alveolar macrophages in porcine lungs (Duan et al., 1997). During viral infections, host biological processes can become inundated from the activity of the viral life cycle, leading to enzymatic changes that alter host metabolic profiles. This in turn can lead to misfunction of macrophage activation signaling that indicates a dysregulated immunologic–metabolic axis (Hicks et al., 2013; Xiao et al., 2015; Langston et al., 2017).

It has been established that susceptibility to PRRSV infection and persistence involves a host genetic component (Dekkers et al., 2017), in which certain genes can behave in either a pro-viral or antiviral manner. Less understood are the actions of small noncoding regulatory and effector RNAs that influence host immunologic and metabolic functions to skew away from homeostasis during PRRSV infection. To begin to understand the effect of small noncoding RNAs (sncRNAs) on the host response to PRRSV, researchers have mostly examined the effects of miRNAs, centered around the canonical functions that miRNAs encompass through the inhibition of gene expression by transcriptional suppression of mRNA and the ability to be both pro- and antiviral (Bruscella et al., 2017). In order to identify and classify other host sncRNA classes affected by PRRSV infection, Fleming and Miller (2018) examined the landscape of porcine whole blood sncRNA. The study revealed whole blood to be a rich landscape of multiple sncRNA, in which to examine the regulatory changes within the host. The current study follows up on the results of Fleming and Miller (2018) using the same dataset with updates to the methods from the previous study to allow for transcriptomic analysis. The current study examined the differential expression of both miRNAs and tRNAs and the effect their expression has on host homeostasis during highly pathogenic type 2 PRRSV infections by examining the biological pathways in which they belong. The purpose and potential impact of the current study are to give insight into the regulatory functions of sncRNAs involved in host cellular communication and homeostasis and into how they relate to how host immune responses, like pro- and anti-inflammatory cytokine signaling, are tempered during infection with a highly pathogenic type 2 PRRSV strain.

## Methods

### Animals and Sample Collection

PRRSV-free 3-week-old crossbreed pigs (Landrace × Yorkshire × Duroc) were purchased from a USDA-approved vendor (Wilson Farms, Wisconsin). Sample collection consisted of whole blood samples (∼2.5 ml/pig) collected from twenty-eight 9-week-old anesthetized pigs by jugular venipuncture. The piglets were inoculated with either a sham inoculation (prepared from MARC-145 cell culture used to propagate the virus) for the controls (N = 14) (2 ml/pig) or challenged (N = 14) with an infectious cDNA clone of a Chinese highly pathogenic (HP) PRRSV strain isolate rJXwn06 (10^4^ TCID_50_/ml, 2 ml/pig). Whole blood sample collection occurred over several time points consisting of 1, 3, and 8 days post infection (dpi) using PAXgene^®^ tubes. Blood samples were stored at -20 C prior to total RNA isolation and NGS library creation. At the conclusion of the study, the pigs were euthanized in the following manner: the animal was physically restrained for the intravenous administration of a barbiturate (Fatal Plus, Vortech Pharmaceuticals, Dearborn, MI) following the manufacturer label dose (1 ml/4.54 kg). Only 24 samples were used due to four animals succumbing prior to 8 dpi.

### RNA Isolation and Sequencing Library Preparation

Total RNA was extracted from 2.5-ml cryopreserved whole blood samples using the protocol from Fleming and Miller (2018) and a modified miRNA extraction kit protocol optimized according to Taxis et al. (2017) for the PAXgene^®^ miRNA and MirVana miRNA isolation kit^™^ (Thermo Scientific, Wilmington, DE, USA). Optimization of these protocols was done to increase small RNA recovery for downstream library creation. All RNA was globin-depleted to account for high levels of globin transcripts using porcine-specific hemoglobin A and B (HBA and HBB) oligonucleotides based on the procedure from Choi et al. (2014). After extraction and globin reduction, the quality and concentration of the total RNA (N = 24) was checked by NanoDrop using each sample. Prior to library creation, sample quality was checked using the Agilent Bioanalyzer 1000 that showed the total RNA quality ranged from a RIN # of 6.5–9.2, and 260/280-nm concentrations ratios were at or above 2 for all samples after globin reduction prior to library preparation. Following quality control checks, the globin-reduced total RNA samples were then used for library preparation for sncRNA sequence generation. Library creation was carried out on 24 samples using the manufacturer’s protocol for the NEBNext multiplex small RNA library prep kit^®^ with a starting RNA amount of ∼220 ng to ∼1.1 µg. Step one of the protocol, the 3’ SR adaptor ligation, was modified from the manufacturer’s protocol to incubate the samples for 18 h at 16°C to increase ligation efficiency of methylated RNAs. Next, the 5’ SR adaptor was ligated, and hybridization of the reverse transcription primer was performed. Samples were then barcoded using the NEBnext indexes (1–24) to allow for multiplexing prior to PCR amplification of cDNA. Samples were then put through a cleanup step using the Qiagen QIAquick PCR purification kit^®^, then checked for quality using the Agilent Bioanalyzer. Small RNA libraries were not size-selected to allow for the capture of multiple sncRNAs between 18–200 nt. Sequencing was carried out on the Illumina Hiseq 3000^™^ at the Iowa State University genomic sequencing center in Ames, IA to produce a total of one 100-bp single-end read for each of the 24 samples.

### Transcriptomic Analysis

The transcriptomic analysis was accomplished using *in silico* resources within the Galaxy web interface (Blankenberg et al., 2010; Afgan et al., 2016). Quality assessment and control were performed using FastQC and TrimGalore (Martin, 2011) to remove adaptors and barcodes from multiplexing. Reads with a quality score below 38 and length less than 18 or longer than 72 nucleotides were discarded. A total of 24 sequences were generated for downstream analysis. The sequenced reads were mapped to the *S.scrofa* 10.2 reference genome from Ensembl using the Hisat2 (Kim et al., 2015) package with default settings. Annotation of gene counts was performed using FeatureCounts software package (Liao et al., 2014) coupled with an in-house-created sncRNA GTF file. The in-house GTF file was based on annotations from release 21 of miRbase, GtRNAdb using tRNAscan-SE 2.0, Ensembl 84 ncRNA database, and the RTH *S.scrofa* 10.2 ncRNA database. The differential gene expression was calculated using the DeSeq2 package with the dispersion model set as local with all other parameters set at their default values. The differential expression was based on the model ∼Treatment_HP-PRRSV, Control_ + Time_1,3,8_ + Treatment : Time. All reported results are based on the interaction of treatment and time and were considered statistically significant at FDR ≤ 0.15 based on a Benjamini and Hochberg FDR adjustment. No cutoff was used for log_2_FC. Venn diagram analysis was conducted using the website http://bioinformatics.psb.ugent.be/webtools/Venn/ (2017). The 10.2 reference genome was used in lieu of the newer 11.1 version due to a current lack of updates to the miRNAs and tRNAs examined within the study. Data has been deposited in a public repository under GEO accession: GSE121980.

### Pathway and Gene Ontology Analysis

Downstream analysis of miRNA gene and biological pathway targets was carried out using miRBase (Griffiths-Jones, 2006; Griffiths-Jones et al., 2006; Griffiths-Jones et al., 2008; Kozomara and Griffiths-Jones, 2011; Kozomara and Griffiths-Jones, 2014) and the DIANA-TOOLS web portal (Vlachos et al., 2015b). All porcine miRNAs used were mature sequences and were first divided into up- or downregulated groups and converted to their human homolog prior to pathway analysis using the MirPath v.3 tool (Vlachos et al., 2015b). Only human sncRNA homologs and pathways were used in the comparisons. All subsequent pathways and G.O. relate to human molecular functions and biological processes for the networks or genes being targeted. The number of genes targeted for each miRNA list was based on information from the TarBase miRNA database (Vlachos et al., 2015a; Paraskevopoulou et al., 2016). The pathway and G.O. analysis used a statistical significance threshold of q ≤ 0.05 based on the Benjamini and Hochberg FDR adjustment for multiple gene set corrections.

### Noncoding RNA Interaction Analysis

The noncoding RNA (ncRNA)–protein interaction analysis was based on conversion of tRNAs to their human homologs. Swine tRNAs were matched using BLASTN to find matching human sequences and gene names prior to using the RAIN module of the RTH database site to predict possible interactions of other ncRNAs and/or mRNAs (Junge et al., 2017). Predicted interactions were presented within STRING DB (Szklarczyk et al., 2015) using the evidence view and have a confidence score of 0.2 or higher with a maximum of 10 predicted interactors allowed.

## Results

### Clinical Evaluation of Infection

**Figure 1:**
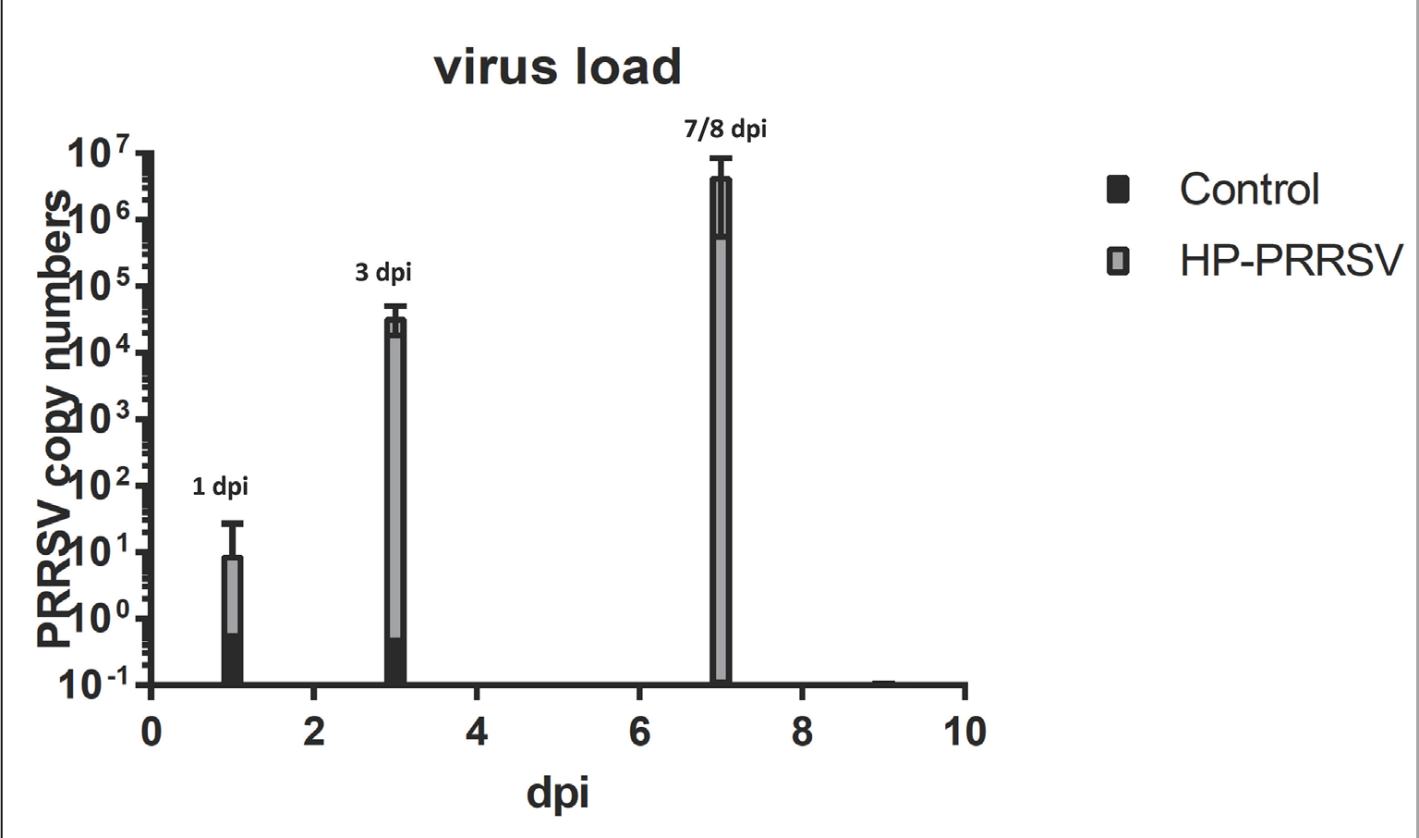
Plot of viral load over time. The graph shows that HP-PRRSV replication increased across the course of infection. The largest increase takes place between 1 and 3 dpi that tracks with large changes in miRNA expression.

Clinical evaluation of the infected animal was examined through an analysis of viral titers. The analysis showed that within the treated samples, viral titers were present at 1 dpi and increased at every time point indicating the success of the HP-PRRSV to replicate. The viral titers for the control samples were considered to be zero, as no replication was detectable (**Figure 1**).

### miRNA and tRNA Differential Expression

The outcome of our study provided differentially expressed (DE) miRNA and tRNA totals for each dpi that were statistically significant at fdr of q ≤ 0.15 except the 1-dpi tRNA results. For the miRNAs, we observed 41 in total at 1 dpi, 14 in total at 3 dpi, and 33 in total at 8 dpi. For the tRNAs, we observed no statistically significant differential expression for 1 dpi and a total of 20 and 40 for 3 and 8 dpi, respectively.

**Table 1A** shows the list of statistically significant tRNAs from the interaction of treatment and time. The tRNA differential expression increased in both magnitude and count as dpi increased. The most abundant tRNA amino acid was alanine (n = 8) at 3 dpi with seven of the eight DE alanine tRNA genes being downregulated (**Supplementary Table 1**). The trna1668_ValAAC (log_2_FC = 2.07) was the most upregulated, and trna1503_ProAGG (log_2_FC = −1.78) was the most downregulated. From 3 dpi, trna1668_ValAAC is predicted to form interaction networks with Surfactant protein B (*SFTPB*), which has a homeostatic effect, as it can support alveolar functions by fostering stability within peripheral air spaces (Whitsett et al., 2010) and the gene Transient receptor potential cation channel, subfamily V, member 1 (*TRPV1*) that is involved in the intercession of inflammatory stressors such as pain (Wang et al., 2018). Also at 3 dpi was the trna202_LeuTAG (log2FC = 1.28), which forms predicted interactions with Toll-like receptors 2 and 4 (*TLR2* and *TLR4*), involved in the monocytic cell ability to recognize antigens and signal pro-inflammatory cytokines and the gene beta-1,4-N-acetyl-galactosaminyl transferase 2 (*B4GALNT2*), also known as *B4GALT*, which is a member of the Beta 4-glycosyltransferase gene family related to the gene beta-1, 4 Galactosyltransferase V (*B4GALT5*) that is shown to also be a target of the multiple miRNAs differentially expressed during this study (Jenuth, 2000; Junge et al., 2017; The UniProt, 2017) (**Figure 1**).

**Table 1:** Ten most differentially expressed tRNAs and miRNAs by dpi. **(A)** tRNA. **(B)** miRNA.

**(A)** | Ten most differentially expressed tRNAs by dpi.			
**DPI**	**GeneID**	**tRNA ID**	**log_2_(FC)**
1	NONE	NONE	NONE
**DPI**	**GeneID**	**tRNA ID**	**log** **_2_** **(FC)**
3	trna1668	ValAAC	2.07
3	trna1634	LeuCAA	1.52
3	trna660	TrpCCA	1.47
3	trna201	CysGCA	1.40
3	trna202	LeuTAG	1.28
3	trna1647	AlaCGC	−1.27
3	trna1245	ProTGG	−1.37
3	trna1635	AlaAGC	−1.58
3	trna1637	AlaCGC	−1.75
3	trna1503	ProAGG	−1.78
**DPI**	**GeneID**	**tRNA ID**	**log_2_(FC)**
8	trna783	GlyGCC	2.37
8	trna630	GlyCCC	1.94
8	trna658	ProCGG	1.89
8	trna1245	ProTGG	1.86
8	trna1270	TyrGTA	1.84
8	trna1665	GlnCTG	−1.57
8	trna1243	HisGTG	−1.61
8	trna648	GlnCTG	−1.66
8	trna453	LysCTT	−1.76
8	trna552	AlaAGC	−1.83
**(B)** | Ten most differentially expressed miRNAs by dpi.			
**DPI**	**GeneID**		**log_2_(FC)**
1	ssc-miR-664-5p		1.21
1	ssc-miR-1306-5p		1.10
1	ssc-miR-145-5p		1.04
1	ssc-miR-296-5p		1.03
1	ssc-miR-18a		0.95
1	ssc-miR-192		−1.05
1	ssc-miR-144		−1.09
1	ssc-miR-148b-3p		−1.28
1	ssc-miR-142-3p		−1.38
1	ssc-miR-374b-5p		−1.57
3	ssc-miR-142-3p		1.17
3	ssc-miR-7		1.08
3	ssc-miR-1839-5p		0.92
3	ssc-miR-21		0.80
3	ssc-miR-142-5p		0.79
3	ssc-miR-125b		−0.76
3	ssc-miR-139-5p		−1.01
3	ssc-miR-99a		−1.18
3	ssc-miR-27b-3p		−1.91
3	ssc-miR-7134-5p		−2.23
8	ssc-miR-145-5p		1.50
8	ssc-miR-10b		1.28
8	ssc-miR-27b-3p		1.28
8	ssc-miR-9841-3p		1.28
8	ssc-miR-125b		1.23
8	ssc-miR-744		−1.17
8	ssc-let-7e		−1.20
8	ssc-miR-1285		−1.33
8	ssc-miR-129a-5p		−1.35
8	ssc-miR-296-3p		−1.59

All miRNA and tRNA log_2_FC values were statistically significant based on a FDR of q ≤ 0.15.

**Figure 2:**
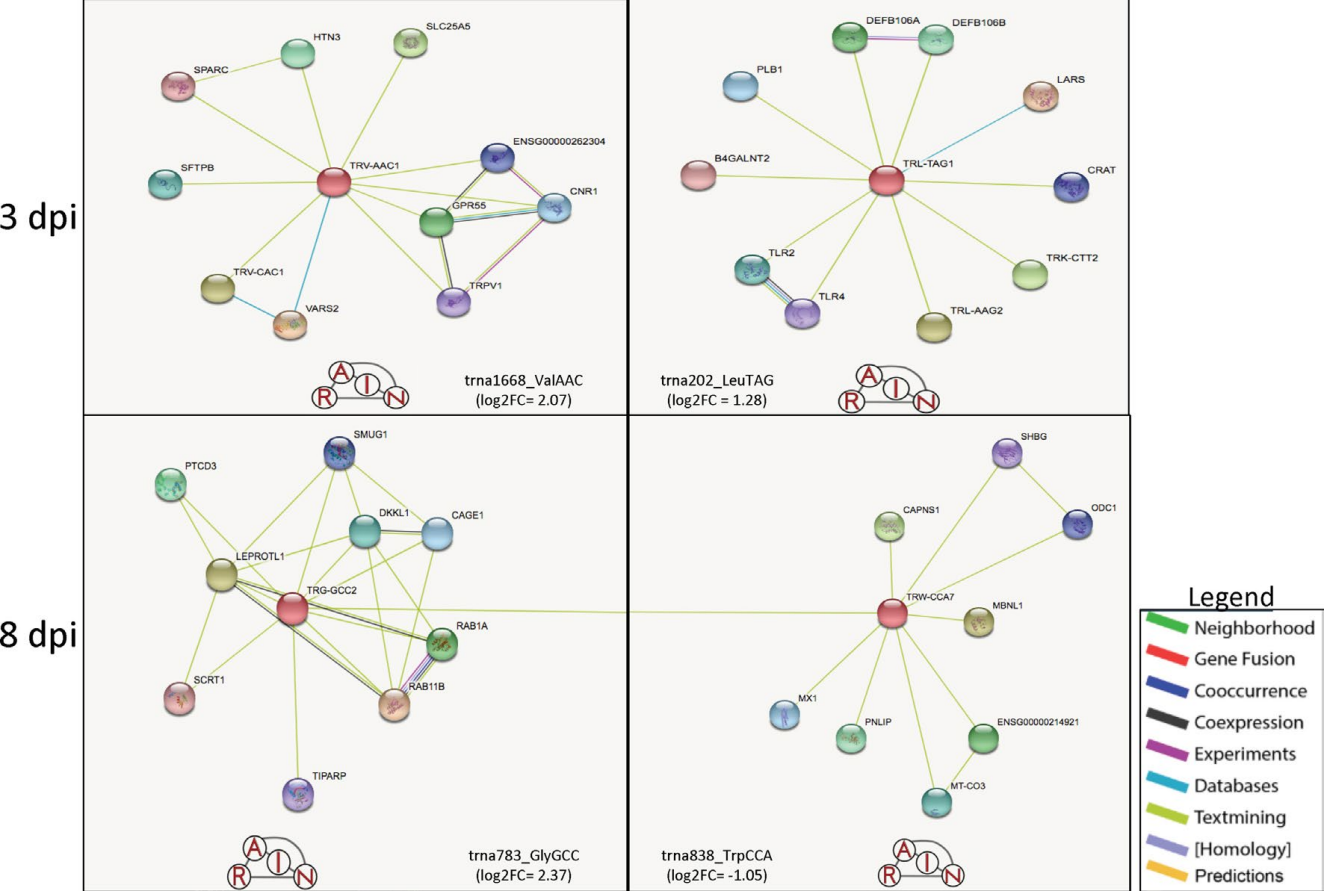
Predicted tRNA:gene interactions for 3 and 8 dpi. The predicted interactions at 3 dpi shows that tRNA_ValAAC could be linked to signaling for inflammation and pain through its connection to *SFTPB* and *TRPV1*, while tRNA_LeuTAG is possibly linked to viral recognition through TLR2 and TLR4 interactions (top). At 8 dpi, tRNA_GlyGCC is predicted to interact with tRNA_TrpCCA, which together are predicted to interact with multiple anti-viral and apoptotic genes such as *TIPARP*, *MX1*, and *CAPNS1*. Figure created using the RAIN software and adapted for inclusion.

For 8 dpi, glycine (n = 10) was the most abundant tRNA amino acid (**Supplementary Table 1**), with trna783_GlyGCC (log_2_FC = 2.37) being the highest upregulated tRNA and trna552_AlaAGC (log_2_FC = −1.83) being the most downregulated (**Table 1A**). The tRNA_GlyGCC is predicted to form interactions with the gene Leptin receptor overlapping transcript-like 1 (*LEPROTL1*), a growth hormone receptor highly expressed in porcine lung tissue (Jenuth, 2000; Demarchi et al., 2007); TCDD-inducible poly(ADP-ribose) polymerase (*TIPARP*), a host defensive gene able to detect mitochondrial damage and bind viral RNA (Kozaki et al., 2017); and RAB1A member RAS oncogene family (*RAB1A*), which is involved in the biological processes of autophagy, IL-8 secretion, and post-translational protein modification (Jenuth, 2000). The tRNA_GlyGCC is also predicted to interact with trna838_TrpCCA (log_2_FC = −1.05) (**Supplementary Table 1**), which is itself predicted to form interactions with the antiviral gene Myxovirus (influenza virus) resistance 1 (*MX1*) and Calpain small subunit 1 (*CAPNS1*), which is involved in the biological processes of autophagy, apoptosis, cell adhesion, and extracellular matrix degradation (Jenuth, 2000; Demarchi et al., 2007; The UniProt, 2017) (**Figure 2**). **Table 1B** shows a list of the top 10 most differentially expressed miRNAs from each dpi, some of which have been implicated as host immunomodulators during PRRSV infections as well as other viral and nonviral (Podolska et al., 2012; Maes et al., 2016; O’Leary et al., 2016; Rosenberger et al., 2017) affronts to the homeostatic state of the porcine host. The most common differentially expressed miRNAs shared by all dpi were ssc-miR-125b (upregulated at 1 and 8 dpi and downregulated at 3 dpi) and ssc-miR-361-3p (all upregulated) (**Supplementary Table 1**).

In relation to previous PRRSV studies of sncRNA, we saw a differential expression across multiple dpi for miRNAs such as ssc-miR-142-3p (1 and 3 dpi), predicted to target the gene guanylate binding protein 5 (*GBP5*), which is shown to harbor a variant that confers some resistance to lowly pathogenic PRRSV strains (Koltes et al., 2015), and also ssc-miR-125b (3 and 8 dpi), an miRNA with anti-viral properties specific to PRRSV (Wang et al., 2013). Our study also uncovered miRNA kinetics that occurred during the 8-day experiment that were novel to the interaction of HP-PRRSV and its host *in vivo*. In upregulated miRNAs such as ssc-miR-664-5p (1 dpi), which has previously shown to become upregulated in bacterially infected lung samples (Podolska et al., 2012), ssc-miR-145-5p (8 dpi) has been revealed to be a potent inhibitor of inflammatory cytokines through its targeting of *CD40*, can prime macrophages in a M2-like manner, and is implicated in reducing lung inflammation in humans suffering from COPD (Guo et al., 2016; O’Leary et al., 2016; Shinohara et al., 2017; Yuan et al., 2017). There was also ssc-miR-144 (1 dpi), which has been indicated as an inhibitor of the anti-viral response to influenza, another major viral respiratory disease that affects swine. There is also evidence that mature form of miR-144 also functions to suppress autophagy within host macrophages (Guo et al., 2017; Rosenberger et al., 2017). Likewise, discerned during this study were 2 miRNA groupings, the miR-30 and the miR-142 families of miRNAs that stood out across the dpi that appeared to be of importance. The miR-30 family of miRNAs appeared at least once on every dpi and was downregulated at all time points except 3 dpi, while the miR-142 family appeared downregulated at 1 and 2 upregulated at 3 and 1 at 8 dpi (**Table 1**) (**Supplementary Table 1**).

### Overall Common KEGG Pathways Analysis

The pathway and G.O. analysis were examined for results that were unique to either upregulated miRNAs or downregulated miRNAs (**Table 2**) or shared between the two (data not shown). The results for the 1-, 3-, and 8-dpi infected samples showed statistical significance (q ≤ 0.05) for multiple G.O. terms and KEGG pathways involved in the maintenance of the extracellular matrix and receptor interactions as well as various immune function-related pathways. Overall, the most prevalent shared pathway was the TGF-beta signaling pathway (*hsa04350*), a pro-inflammatory pathway, which was targeted on all dpi by the up- and downregulated miRNAs. The second most common pathway was the proteoglycans in cancer pathway (*hsa05205*). The proteoglycans in cancer pathway consisted of four total pathways: hyaluronan (*HA*), chondroitin sulfate/dermatan sulfate (*CSPG/DSPG*), keratan sulfate (*KSPG*), and heparan sulfate (*HSPG*). This composite of proteoglycan pathways is involved in structural integrity, extracellular matrix, and receptor-ligand binding and appeared as one of the most statistically significant pathways for each dpi as either a common (3 and 8 dpi) or unique (1 dpi) pathway. Other pathways shared across multiple dpi by both up- and downregulated miRNAs included endocytosis (*hsa04144*), bacterial invasion of epithelial cells (*hsa05100*), adherens junction (*hsa04520*), and mTOR signaling pathway (*hsa04150*).

### miRNA Pathway Target Prediction and G.O. Analysis

The pathways that were targeted by the upregulated vs. downregulated miRNAs (**Table 2**) allowed us to observe which functional and biological processes were possibly being altered by the inhibition or activation of genes involved in these processes within the host. At 1 dpi, the unique pathways targeted by the upregulated miRNAs were mostly key structural and signaling KEGG pathways that would appear to assist viral entry. This included pathways such as focal adhesion (*hsa04510*), proteoglycans in cancer pathway (*hsa05205*), and the notch signaling pathway (*hsa04330*). In regards to the downregulated miRNAs expressed at 1 dpi, the unique pathways included key immune-related KEGG pathways related to inflammatory immune functions during PRRSV infection. This included traditional innate immune response pathways such as the TNF signaling pathway (*hsa04668*), NF-kappa B signaling pathway (*hsa04064*), the AMPK signaling pathway (*hsa04152*) capable of sensing metabolic stressors to host homeostasis, and the multifunction ECM–receptor interaction (*hsa04512*) pathway (Jenuth, 2000; Kanehisa et al., 2016; Kanehisa et al., 2017; The UniProt, 2017).

At 3 dpi, a switching of expression profiles was observed. For the upregulated miRNAs, there was an increase in the number of immune-related KEGG pathways that were targets of inhibition observed at this time point. Pathways included in this group were: NF-kappa B signaling pathway (*hsa04064*) and ECM–receptor interaction (*hsa04512*) that were activated on the previous dpi, and the PI3K-Akt signaling pathway (*hsa04151*) involved in metabolism and apoptosis. The theme of the pathways unique to the downregulated miRNAs also appeared to switch at 3 dpi. For the downregulated miRNAs, emphasis switched from mostly immune regulated to a higher quantity of structural-related pathways with roles in viral entry of respiratory tissue types such as focal adhesion (*hsa04510*), Mucin type O-Glycan biosynthesis (*hsa00512*), and Glycosaminoglycan biosynthesis—keratan sulfate (*hsa00533*) (Jenuth, 2000; Souza-Fernandes et al., 2006; Kanehisa et al., 2016; Kanehisa et al., 2017; The UniProt, 2017). By 8 dpi, the pathways being targeted by the up- and downregulated miRNAs continued to display very kinetic profiles. The upregulated miRNAs now targeted pathways that involved both key structural- and immune-related functions, while the downregulated miRNAs targeted pathways continued to place emphasis again on host immune functions like apoptosis (*hsa04210*) and the Wnt signaling pathway (*hsa04310*) (Pecina-Slaus, 2010; Kanehisa et al., 2016; Villasenor et al., 2017) (**Table 2**).

Additionally, a gene ontology (G.O.) analysis was conducted for the upregulated miRNAs in order to facilitate a better understanding of what biological and molecular functions were being inhibited by the overexpression of the different miRNAs. A Venn diagram (not shown) was used to filter the G.O. terms and determine which were common or unique to each time point in an attempt to elucidate which biological functions of the host were the most attenuated by miRNA overexpression. The terms that were shared across all time points (n = 127) included a majority of host immune processes generally related to viral–host interactions during infections. The shared list contained the G.O. terms negative regulation of type I interferon production (GO:0032480), extracellular matrix disassembly (GO:0022617), and virus receptor activity (GO:0001618).

At 1 dpi, the unique G.O. terms (n = 20) included antigen processing and presentation of peptide antigen *via* MHC class I (GO:0002474), ncRNA metabolic process (GO:0034660), negative regulation of transforming growth factor beta receptor signaling pathway (GO:0030512), and tRNA metabolic process. At 3 dpi, the counts of unique terms dropped considerably (n = 6) from 1 dpi and highlighted terms related to sensing of biotic and abiotic stressors and inhibition by small RNA functions with the terms cytoplasmic stress granule (GO:0010494) and negative regulation of translation (GO:0017148). The unique G.O. terms associated with the miRNA upregulation at 8 dpi (n = 41) were the largest grouping of terms of all time points and encompassed multiple host immune and metabolic function-related terms. Some of the key terms from 8 dpi included collagen catabolic process (GO:0030574), O-glycan processing (GO:0016266), and type I interferon signaling pathway (GO:0060337). The G.O. terms that were unique to the downregulated miRNAs at each dpi were also examined and showed groupings of terms that indicated targeting of various immune functions (not shown).

## Discussion

### Interaction of Treatment and Dpi Reveals Pro-Viral and Anti-Viral Battle Over Host Pathways

Examination of the results indicated that the miRNAs and tRNAs displayed expression patterns that seem to suggest they behave in a manner that both promotes and fights HP-PRRSV infection. This battle for post-transcriptional control over the regulation of host gene expression during infection appeared to take place on several fronts. These fronts were highlighted by the differential expression of candidate miRNAs, tRNAs, and pathways related to several different host biological groupings. The groupings are best represented by the relationship of the pathways they contain, such as structural-related networks that consist of pathways that affect structural integrity and receptor binding. Additionally, there are the immune function-related pathways that spotlight the tug-of-war between host and pathogen. Lastly, there are the pathways that control metabolic activity, which collectively point out the perturbation the virus causes to host homeostasis. Even more striking is that these biological groupings are unique to either the overexpressed or under-expressed miRNAs and reinforced by the ncRNA:mRNA interactions predicted for some of the tRNA genes (Banerjee et al., 2013; Bai et al., 2015).

### HP-PRRSV Infection Stimulated Differential Expression of Key Extracellular miRNAs

Analysis of the miRNA results yielded a group of candidates from each dpi that appeared to be linked to the immunosuppressive effects of the HP-PRRSV infection. Many of these were upregulated miRNAs listed as residing in the extracellular space of cells and may possibly highlight the usurping of host extracellular miRNAs by HP-PRRSV to facilitate viral entry/replication. The upregulation was matched by downregulation of additional miRNAs, likely still under host control, which could help boost the activity and expression of immune-related pathways. The direction of expression for the miRNAs were closely tied to the dpi, as many were observed to be overexpressed at one time point, only to be under-expressed at another. Despite this, the results yielded candidate miRNA’s with potential anti- and pro-viral actions during HP-PRRSV infections of swine. From the upregulated miRNAs at 1, 3, and, 8 dpi were miRNAs Ssc-miR-145-5p, Ssc-miR-142-3p, and Ssc-miR-125b, respectively. The miRNA Ssc-miR-125b (upregulated at 1 and 8 dpi only) along with Ssc-miR-361-3p (upregulated at all dpi) were the only miRNAs to appear statistically significant at each dpi. The miRNA Ssc-miR-145-5p has been shown to be an inhibitor of inflammatory cytokines signaling with the ability to cause alternative macrophage priming that accents anti-inflammatory signaling within the host (Guo et al., 2016; Yuan et al., 2017). This may be evidence of an early ability of PRRSV to suppress innate immune functions or signaling by overriding the M1 priming that would initiate host pro-inflammatory signaling. Also, of interest at 1 dpi was the downregulated Ssc-miR-144, which in its mature form can suppress proper macrophage functions and is also considered an inhibitor of the anti-viral response to influenza, another major porcine respiratory disease (Rosenberger et al., 2017). The difference in the previously studied functions of Ssc-miR-145-5p and Ssc-miR-144 show that at 1-dpi expression of the miRNAs is gauged in both pro- and anti-viral directions.

Upregulated at 3 dpi was the miRNA Ssc-miR-142-3p, which specifically when upregulated has been shown to impair the ability of monocyte-derived cells to properly present and process antigens to the adaptive arm of the immune system (Naqvi et al., 2016). This gives insight into a possible mechanism that HP-PRRSV uses for stalling the host adaptive immune response. Also observed in the study were other differentially expressed members of the miR-142 family of miRNAs. Also upregulated at 3 dpi was the related Ssc-miR-142-5p, which is predicted to target porcine guanylate-binding protein 5 (*GBP5*) gene, which contains the WUR SNP, a low pathogenic PRRSV resistance variant (Koltes et al., 2015). The 3-dpi overexpression of these miRNAs may be evidence of possible mechanisms available to HP-PRRSV strains to stall host immune response and possibly cancel out any protection offered by the WUR variant through GBP5 silencing.

The upregulation of miRNAs at 1 and 3 dpi seemed to favor pro-viral activity within the host; however, by 8 dpi, miRNA upregulation was more anti-viral. This was observed in the host overexpression of Ssc-miR-125b, an extracellular miRNA that is computationally predicted to limit PRRS viral levels through suppressive targeting of host NF-kB signaling (Qureshi et al., 2014). These phenomena may be related to the active role the miRNA plays in the regulation of monocytic cell inflammatory signaling (Duroux-Richard et al., 2016).

### tRNA Differential Expression During HP-PRRSV Infection Indicates Change in Host Homeostasis Benefitting HP-PRRSV

The tRNA differential expression was not significant at 1 dpi but was statistically significant at 3 and 8 dpi after HP-PRRSV infection. Interestingly, the pattern of statistically significant tRNAs and their magnitudes of expression (**Supplementary Table 1**) appear to follow the trend seen in the viral load that increased with dpi (**Figure 1**). The changes in tRNA expression overtime are possibly the result of the virus modulating tRNA expression through hijacking host cellular resources. This change in host resources can then lead to changes in the metabolism of the host that eventually disrupts the proper activation of monocytic cells such as macrophages, allowing the virus to proceed unimpeded by the host cytokines (Langston et al., 2017) toward entry and proliferation. In a 2016 paper, Rappe et al. (2016) was able to observe that PRRS type 1 and 2 virus nucleocapsids can contain an amino acid substitution that replaces a threonine with alanine causing the PRRS virus strain in that study to escape immune system protection. Therefore, it is possible that the virus is promoting differential expression of host alanine as a means of both hijacking needed nutrients and avoiding detection by innate immunity. There is also a possibility that the consistent downregulation of tRNA alanine genes at 3 and 8 dpi (**Supplementary Table 1**) is host initiated to hasten cellular degradation to limit host resources to the virus.

The trna1668_ValAAC was predicted to form interaction networks with *SFTPB* and *TRPV1* (**Figure 2**), two genes involved in processing host signals for inflammation and pain that could also be linked to the changes in miRNA expression at 3 dpi that are shifting the host immune response. Additionally, at 3 dpi is the predicted interaction of the trna202_LeuTAG with the Beta 4-glycosyltransferase gene family that also contains genes involved in the functioning of chondroitin, a major proteoglycan pathway shown to be highly targeted by the miRNAs at each dpi. This can be seen in the results at 3 dpi where miRNAs ssc-miR-27b-3p and ssc-miR-23a-3p targeting *B4GALT5* (Zhang et al., 2018) are being downregulated in the mucin type-o pathway in a possible attempt to bolster mucosal immunity in the host airway.

The prevalence of tRNA glycine genes at 8 dpi could be linked to the role glycine plays in the biochemical composition of collagen (Li and Wu, 2018), a tissue type composed of genes such as *COL4A1* and *COL5A2* shown in previous studies to be dysregulated during HP-PRRSV infections (Miller et al., 2017), and targeted by upregulated miRNAs in different pathways during our study (**Table 2**). Glycine is one of the key components in collagen, and collagen is a key component of the ECM, which our results showed to be compromised by multiple upregulated miRNAs (**Table 1**) (**Supplementary Table 1**). Additionally, glycine and its metabolites are needed by mammals like swine to support proper immune functions. However, the upregulation at 8 dpi could be more closely related to glycine’s ability as an anti-inflammatory molecule.

**Table 2:** Unique KEGG pathways targeted differentially expressed miRNAs.

DPI	KEGG pathways targeted by upregulated miRNAs	Key targets within pathways	Classification
1	*Focal adhesion* (hsa04510)	*CAV2, AKT1, COL4A1, SHC3*	Structural
1	*Proteoglycans in cancer* (hsa05205)-4 total pathways	***Hyaluronan(HA)*** * = AKT1, SLC9A1; * ***Chondroitin sulfate/dermatan sulfate(CSPG/DSPG)*** * = THBS1, MMP9, CD63, AKT1, EGFR; * ***Keratan sulfate(KSPG)*** * = SMAD2, MDM2, TP53, LUM; * ***Heparan sulfate(HSPG)*** * = MMP9, ITGAV, AKT1, EGFR, ITGA5, VEGFA*	Structural
1	*FoxO signaling pathway* (hsa04068)	*EGFR, PTEN, AKT1, BCL6, ATM*	Immune/Metabolism
1	*p53 signaling pathway* (hsa04115)	*ATM, TP53, PTEN,*	Immune
1	*Adherens junction* (hsa04520)	*EGFR, MET, CTNNB1*	Structural
1	*Notch signaling pathway* (hsa04330)	*ADAM17,*	Homeostatic
3	*PI3K-Akt signaling pathway* (hsa04151)	*AKT3, OSMR, PTEN, PIK3CD*	Immune/Metabolism
3	*B cell receptor signaling pathway* (hsa04662)	*BCL10, NFATC3, IKBKB, NFKBIA, NFKB1*	Immune
3	*ECM–receptor interaction* (hsa04512)	*THBS1, TNC, ITGAV, CD44, CD47*	Structural/ Immune
3	*NF-kappa B signaling pathway* (hsa04064)	*IRAK1, PTGS2, BCL10, XIAP, NFKB1, NFKB2, NFKBIA, IKBKB, TAB2*	Immune
8	*Fc gamma R-mediated phagocytosis* (hsa04666)	*AKT1, INPPL1, DNM2, CFL2, MAPK1*	Immune
8	*MAPK signaling pathway* (hsa04010)	*PDGFRB, AKT1, NFATC3, TP53, TAB2, MAP3K7, MAPK1*	Immune
8	*ECM–receptor interaction* (hsa04512)	*TNC, THBS2, ITGB1, ITGAV, FN1, LAMC1*	Structural/ Immune
DPI	KEGG pathways targeted by downregulated miRNAs	Key targets within pathways	Classification
1	*ECM–receptor interaction* (hsa04512)	*THBS2, CD44, CD47, ITGAV*	Structural/ Immune
1	*B cell receptor signaling pathway* (hsa04662)	*CD81, VAV3, IFITM1, NFATC3, BCL10, MALT1, CHUK*	Immune
1	*AMPK signaling pathway* (hsa04152)	*PRKAG2, PFKFB3, MAP3K7, TSC1, MTOR, ULK1*	Metabolism
1	*NF-kappa B signaling pathway* (hsa04064)	*TLR4, IL1B, VCAM1, PTGS2, BIRC2, NFKB1, ICAM, BCL2, TNFAIP3*	Immune
1	*Fc gamma R-mediated phagocytosis* (hsa04666)	*VAV3, DOCK2, PTPRC, INPPL1, DNM2*	Immune
1	*VEGF signaling pathway* (hsa04370)	*VEGFA, PTGS2, PTK2,*	Signaling
1	*TNF signaling pathway* (hsa04668)	*SOCS3, IL1B, IL6, TNFAIP3, BIRC2, VCAM1, PTGS2, MMP9, CSF1, CXCL10, ATF4, LIF*	Immune
1	*Apoptosis* (hsa04210)	*TNFSF10, CAPN2, CASP3, IL1B, IL1R1*	Homeostatic/Metabolism
1	*Influenza A* (hsa05164)	*XPO1, RSAD2, TLR4, SOCS3, TBK1, IRF3*	Immune
3	*Focal adhesion* (hsa04510)	*VEGFA, PTK2, PDPK1*	Structural
3	*Fc gamma R-mediated phagocytosis* (hsa04666)	*ARF6, MARCKS, MAPK1, WASF2*	Immune
3	*AMPK signaling pathway* (hsa04152)	*ADIPOR2, PRKAA1, FOXO3, HMGCR, PDPK1, TSC1*	Metabolism
3	*Glycosaminoglycan biosynthesis - keratan sulfate* (hsa00533)	*B4GALT1*	Structural
3	*Mucin type O-Glycan biosynthesis* (hsa00512)	*B4GALT5, GCNT1, C1GALT1C1*	Metabolism
3	*HIF-1 signaling pathway* (hsa04066)	*HIF1A, CAMK2D*	Homeostatic
8	*Glycosaminoglycan biosynthesis—keratan sulfate* (hsa00533)	*B4GALT1*	Structural
8	*VEGF signaling pathway* (hsa04370)	*VEGFA, KDR, AKT2, PTGS2*	Signaling
8	*Wnt signaling pathway* (hsa04310)	*WNT9A, PLCB4, CTNNB1,*	Immune
8	*Apoptosis* (hsa04210)	*BCL2, AKT2, TNFSF10, IL1A, MYD88, FADD, IRAK1*	Homeostatic/Metabolism
8	*PI3K-Akt signaling pathway* (hsa04151)	*PTEN, AKT2, F2R, IL6R, IRS1, FOXO3*	Immune/Metabolism
8	*Glycosaminoglycan biosynthesis - heparan sulfate / heparin* (hsa00534)	*EXTL3, NDST2*	Structural
8	*HIF-1 signaling pathway* (hsa04066)	IL6R, STAT3, VHL, CUL2, LTBR, IL6, TLR4	Homeostatic

The upregulated miRNAs targeted genes within the pathways for inhibition. This inhibition affected mostly structural pathways at 1 dpi, immune functions at 3 dpi, and a combination of both at 8 dpi. The downregulated miRNAs targeted genes for activation within the pathways. The activated pathways were mostly involved in immune functions at 1 and 8 dpi and structural integrity at 3 dpi. Key pathway targets based on miRNA targets within listed KEGG pathways. Analysis of statistical significance threshold set at an FDR of q ≤ 0.05 for all pathways listed.

### KEGG Pathways Analysis Indicates Coupled Viral Entry and Immunosuppressive Effect of HP-PRRSV

**Figure 3:**
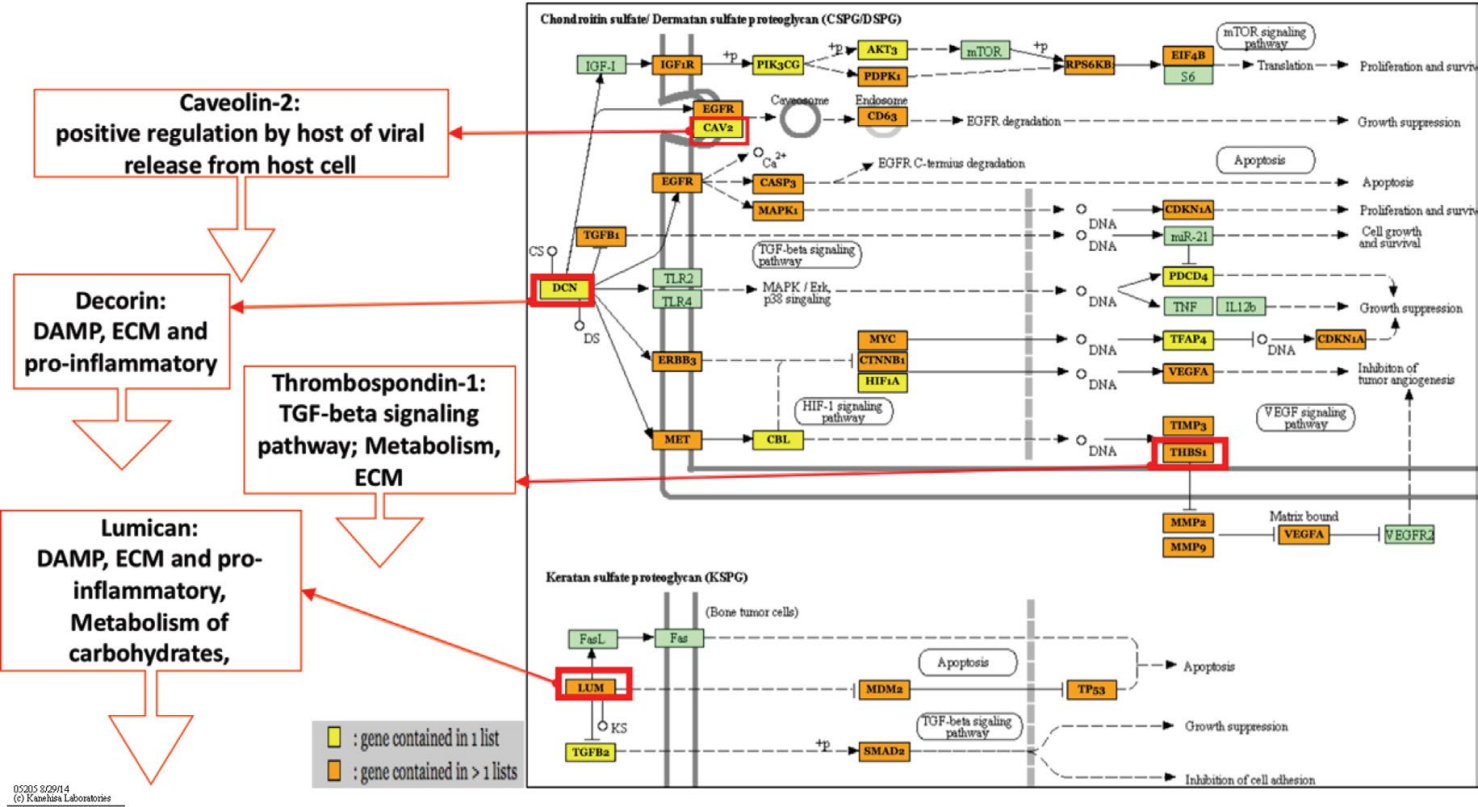
Proteoglycans in cancer-predicted pathway. This figure highlights two of the four proteoglycans in cancer pathways, chondroitin sulfate/dermatan sulfate (*CSPG/DSPG*) and keratan sulfate (*KSPG*). These networks show that there are multiple genes (orange and yellow boxes) related to cytokine signaling and viral entry that are being targeted for inhibition that would cause host dysregulation and impair the ability to properly respond or maintain homeostasis during infection. Additionally, genes shown in previous HP-PRRSV transcriptome studies to be downregulated are shown to be targets of the upregulated miRNAs. *DCN* and *LUM* are DAMPs, which lead to downregulation of inflammatory DAMP signals and downstream *TGF-Beta* signaling, which is involved in anti-viral immunity. **Figure 3** shows upregulated miRNAs only from all time points. Figure created using Mirpath V3 software and adapted for inclusion. Figure based on KEGG pathways. Figure legend refers to if gene in pathway has 1 (yellow) or >1 (orange) miRNAs targeting it.

The pathways that were shared across all dpi such as the TGF-beta signaling pathway is a continuation of the suppression observed with the four proteoglycans in cancer pathways. The DE miRNAs within the TGF-beta signaling pathway likely perturbs multiple transcription and cofactors leading to immunosuppression, delayed apoptotic induction, and ECM dysregulation that is also shown to be affected. The first front of the battle between host and virus appears to take place at 1 dpi within inhibited structural pathways, concomitant with viral entry and proliferation, and activated immune response pathways that support pro-inflammatory signaling (**Table 2**). The other common pathway, the proteoglycans in cancer pathway, was actually unique to 1 dpi (**Figure 3**) and revealed a class of miRNA-targeted structural genes that also functions as part of the innate immunity collectively referred to as damage-associated molecular pattern signals or DAMPs. Two DAMPs in particular, decorin (*DCN*) and lumican (*LUM*), have been shown to be heavily differentiated in HP-PRRSV infections (Miller et al., 2017) in a previous study and were targets of multiple upregulated miRNAs within our results. It is possible that miRNA overexpression reduces the ability of proteoglycan DAMPs like *DCN* and *LUM* to promote inflammatory cytokine signaling (Merline et al., 2011; Moreth et al., 2012), while also weakening the ECM to help promote viral invasion and proliferation.

The pathways targeted for miRNA-directed suppression at 1 dpi such as focal adhesion and the proteoglycans in cancer pathway could indicate early viral manipulation of host resources linked to documented viral entry strategies involving the binding of host glycoprotein molecules, which have many identities such as cell-surface and transmembrane receptors that make up extracellular matrix proteins such as proteoglycans and integrins (Rabinovich et al., 2012; Cossart and Helenius, 2014). These pathways are crucial to the makeup of the extracellular matrix and may indicate why the ECM–receptor interaction (*hsa04512*) pathway is specifically targeted for activation by the downregulated miRNAs. In line with the viruses’ infection and proliferation at 1 dpi, the host was not only bolstering defense to viral entry with increased ECM–receptor activity but also innate immune responses for cytokine signaling possible through activity of the TNF signaling pathway (*hsa04668*) and the NF-kappa B signaling pathway (*hsa04064*). There is also some indication that the host metabolism is starting to become perturbed at 1 dpi due to activation of genes within the AMPK signaling pathway (*hsa04152*) that is involved in metabolic functions, especially in low energy states (Mihaylova and Shaw, 2011).

By 3 dpi, the unique up- and downregulated miRNAs had switched targeting roles with respect to the biological processes that appeared to be either inhibited or activated within the host. The upregulated miRNA was now inhibiting immune function pathways such as the ECM–receptor interaction pathway (*hsa04512*) and NF-kappa B signaling pathway (*hsa04064*) that were previously being activated. The switch from structural to immune pathway inhibition may serve as possible indicators of the dysregulation of monocytic cell immune functions experienced by infected macrophages to protect and prolong viral replication. Also supporting the idea that HP-PRRSV had switched to modulating host immune pathways to support survival was the observance of the PI3K-Akt signaling pathway (*hsa04151*) inhibition. This pathway is known to be involved in cell survival and can be usurped by viral pathogens to promote survival (Dunn and Connor, 2012); therefore, the host may be regulating miRNA expression against the pathway in an attempt to reduce usurpation by HP-PRRSV. Another suggestion is that the inhibition of the PI3K-Akt signaling pathway could be linked to the 1-dpi inhibition of the FoxO signaling pathway (*hsa04068*) and is a means for HP-PRRSV to lower the apoptotic functions for these pathways to promote self-survival against host immune functions. The unique pathways based on the downregulated miRNAs at 3 dpi had now begun to target more structural and metabolic pathways such as the Mucin type O-Glycan biosynthesis (*hsa00512*), AMPK signaling pathway (*hsa04152*), and the HIF-1 signaling pathway (*hsa04066*). It is uncertain whether this potential increase in structural and metabolic activity is anti- or pro-viral in nature; however, it does show evidence that host homeostasis is being dysregulated. If it is anti-viral in nature, the pathway activity may be supporting repair of components such as the ECM after viral entry. However, if it is pro-viral, it might hint at AMPK and HIF-1 signaling pathway involvement in metabolic processes that can detract from normal macrophage activation and may be connected to the 8 dpi upregulation of glycine tRNAs based on *HIF1A*’s role in glycolysis (Langston et al., 2017).

By 8 dpi, the affected unique pathways had flipped roles once again in regard to the biological clustering of inhibited and activated pathways. The 8-dpi inhibited pathways now incorporated a combination of structural and immunological pathways that included the ECM–receptor interaction pathway (*hsa04512*), Fc gamma R-mediated phagocytosis (*hsa04666*), and the MAPK signaling pathway (*hsa04010*). The 8-dpi pathway analysis also showed that the unique activated pathways also clustered around both immune and structural pathway indicating some importance of proteoglycans and pro-inflammatory cytokine signaling. It is also possible that the pathways targeted by the miRNA differential expression at 8 dpi is a reflection of the losing battle between host immunity and HP-PRRSV virulence related to impairment of normal macrophage functions due to PRRSV cell tropism.

## Conclusions

Taken together, the pathway analyses suggest that the changes in host homeostasis were affected through the ability of HP-PRRSV to disturb host structural, immunologic, and metabolic pathways. These targeted pathways, along with the predicted tRNA:gene interactions, highlighted both inhibition and activation of pathways involved in viral entry, proliferation, and pro-inflammatory signaling that may underlie the ability of PRRSV to hinder homeostasis through sncRNA dysregulation. Small noncoding RNA (sncRNA) expression during HP-PRRSV infection can affect the differential expression of miRNAs and tRNAs and can exist as an evasion to canonical host immune responses when expressed in patterns exhibited across the experiment’s time points. Highly pathogenic PRRSV appeared to have the ability to induce differential expression of both miRNAs and tRNAs as part of its pathogenic course that perturbed structural, metabolic, and immunogenic pathways. The action of these sncRNAs created post-transcriptional changes to the overall ability of the host to maintain cellular homeostasis in the presence of the pathogen.

## Data Availability

The datasets generated and/or analyzed for this study can be found in the GEO repository, GSE121980, http://www.ncbi.nlm.nih.gov/geo/query/acc.cgi?acc=GSE121980.

## Ethics Statement

The animal use protocol was reviewed and approved by the Institutional Animal Care and Use Committee (IACUC) of the National Animal Disease Center-USDA-Agricultural Research Service. Written informed consent to use the animals in the study was obtained from the Wilson Farms, WI.

## Author Contributions

LM contributed to the study conception, data collection, research design, and manuscript writing. DF contributed to the data preparation, data analysis, research design, and manuscript writing.

## Funding

Mention of trade names or commercial products in this article is solely for the purpose of providing specific information and does not imply recommendation or endorsement by the US Department of Agriculture. USDA is an equal opportunity provider and employer. This work was mainly supported by the USDA NIFA AFRI 2013-67015-21236 and in part by the USDA NIFA AFRI 2015-67015-23216. DF was supported in part by an appointment to the Agricultural Research Service Research Participation Program administered by the Oak Ridge Institute for Science and Education (ORISE) through an interagency agreement between the US Department of Energy (DOE) and the US Department of Agriculture. ORISE is managed by Oak Ridge Associated Universities under DOE contract no. DE-AC05-06OR23100.

## Conflict of Interest Statement

The authors declare that the research was conducted in the absence of any commercial or financial relationships that could be construed as a potential conflict of interest.

## Supplementary Material

Supplementary Table 1 tRNA and mRNA differential expression by dpi. Excel chart of the differential expression (FDR ≤ 0.15) observed for each class of sncRNA for 1, 3, and 8 days post-infection with HP-PRRSV.

Supplementary Table 2 Unique upregulated and downregulated G.O. terms by dpi. Excel chart of statistically significant (FDR ≤ 0.05) G.O. terms based on miRNA analysis split by direction of differential expression and dpi.

Supplementary Table 3 Filtering and mapping statistics based on size selection of reads 18–80 bps after library creation.
